# Compensatory Class III malocclusion treatment associated with mandibular canine extractions

**DOI:** 10.1590/2177-6709.22.6.086-098.bbo

**Published:** 2017

**Authors:** Guilherme Janson, Olga Benário Vieira Maranhão

**Affiliations:** 1 Universidade de São Paulo, Faculdade de Odontologia de Bauru, Departamento de Ortodontia (Bauru/SP, Brasil).

**Keywords:** Canine extraction, Class III malocclusion, Orthodontics, Corrective

## Abstract

Skeletal Class III malocclusions are ideally treated with orthodontic-surgical approaches. However, if there are no significant soft tissue implications and the patient does not want to undergo orthognatic surgery, other treatment options may be considered. The current case report describes a compensatory alternative for Class III malocclusion treatment, by means of mandibular canine extractions. This treatment alternative provided facial profile and occlusal improvement, which remains stable seven years posttreatment.

## RESUMO

As más oclusões esqueléticas de Classe III são idealmente tratadas com intervenções ortodôntico-cirúrgicas. Contudo, se não existirem implicações estéticas faciais e se o paciente não desejar se submeter à cirurgia ortognática, outras opções de tratamento podem ser consideradas. O presente caso clínico descreve um tratamento compensatório alternativo para a má oclusão de Classe III, com extrações de caninos inferiores. Esse tratamento alternativo propiciou melhoras no perfil facial e na oclusão, que permaneceu estável após sete anos da sua finalização.

## INTRODUCTION

Class III is a complex malocclusion that involves dental, skeletal or both structures.^1^ ^,^ ^2^ Treatment usually consists in a compensatory or orthodontic-surgical approach,^3^ but the results are not always predictable. In cases with great skeletal vertical and anteroposterior discrepancies, the orthodontic treatment associated with a surgical approach might be the best treatment plan.^1^ ^,^ ^4^ However, in some cases the patient is more interested in less invasive interventions. In these situations, one option is compensatory treatment with extractions, which also provides good occlusal and acceptable esthetic results, with good stability.^5^ ^-^ ^8^ A compensatory approach is also indicated when the patient does not have esthetic complaints and the anteroposterior skeletal discrepancy is not severe.^9^

Usually, protocols in compensatory orthodontic treatment involve premolar extractions, but incisor and molar extractions are also described in the literature.^8^ ^,^ ^10^ ^,^ ^11^ In this case report, mandibular canine extractions were performed to improve the occlusal relationships and facial esthetics.

## DIAGNOSIS

A 13-year-old female patient was referred for treatment by her parents after many previous orthodontic assessments. The patient had a skeletal Class III malocclusion pattern and previous treatment plans consisted in surgical-orthodontic approaches. However, her parents did not accept a surgical treatment, and searched for a different opinion with the first author. Almost all of her relatives did not present a skeletal Class III malocclusion pattern, except for her paternal grandfather who had a similar pattern. 

**Figure 1:**
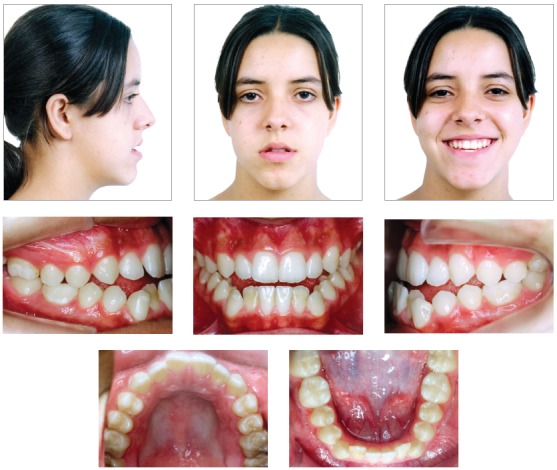
Initial extra- and intraoral photographs.

**Figure 2:**
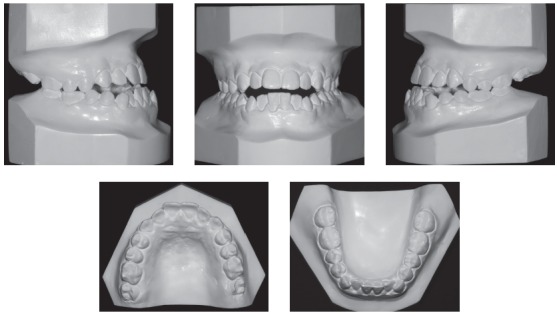
Initial dental models.

**Figure 3:**
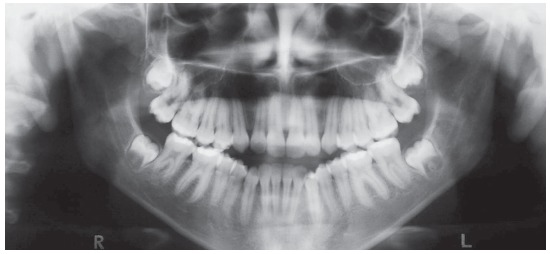
Initial panoramic radiograph.

The extraoral examination showed a skeletal Class III malocclusion pattern, vertical growth, incompetent lip seal, mouth breathing and lingual thrust during speech and swallowing. Intraorally, she presented with a complete bilateral Class III malocclusion, moderate mandibular anterior crowding, mild maxillary anterior crowding, maxillary midline deviated 1.5 mm to the left, anterior open bite, overjet of -1 mm, and tonsils hypertrophy (Figs 1 and 2). The panoramic radiograph shows that all teeth were present, with the third molars under development. No other significant abnormality was found (Fig 3).

**Figure 4:**
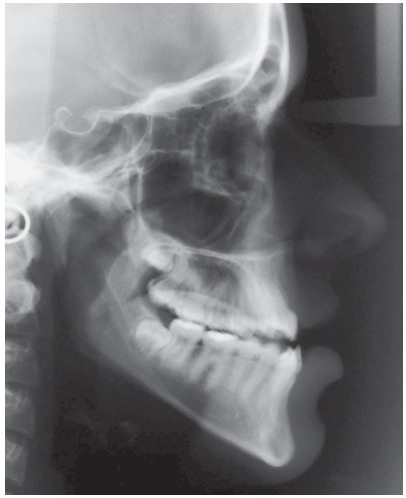
Initial cephalogram.

Cephalometrically, she had a slightly protruded maxilla, mandibular protrusion, a moderate skeletal Class III apical base discrepancy, and an accentuated vertical growth pattern. The maxillary incisors were labially tipped and slightly protruded, and the mandibular incisors were lingually tipped and linearly well positioned (Table 1 and Fig 4).

**Table 1:** Cephalometric status at the initial, final and posttreatment stages.

	Measurements		Normal	A	B	C	Dif. A/B
Skeletal pattern	SNA	(Steiner)	82^o^	84.2^o^	84.8^o^	84.5^o^	-0.6^o^
	SNB	(Steiner)	80^o^	86.1^o^	84.5^o^	83.9^o^	-1.6^o^
	ANB	(Steiner)	2^o^	-1.9^o^	0.2^o^	0.5^o^	+1.7^o^
	Wits	(Jacobson)	♀ 0 ±2 mm ♂ 1 ±2 mm	-9.5 mm	-6.4 mm	-6.7 mm	+3.1 mm
	Angle of convexity	(Downs)	0^o^	-6.4^o^	-4.2^o^	-3.3^o^	+2.2^o^
	Y-axis	(Downs)	59^o^	58.5^o^	58.3^o^	61^o^	-0.2^o^
	Facial angle	(Downs)	87^o^	95^o^	95.6^o^	93.1^o^	+0.6^o^
	SN-GoGn	(Steiner)	32^o^	38.6^o^	39.3^o^	40^o^	+0.7^o^
	FMA	(Tweed)	25^o^	33.3^o^	32.9^o^	35.2^o^	-0.4^o^
Dental pattern	IMPA	(Tweed)	90^o^	73.1^o^	71.3^o^	75.1^o^	-1.8^o^
	1.NA (degrees)	(Steiner)	22^o^	26.3^o^	21.7^o^	18.3^o^	-4.6^o^
	1-NA (mm)	(Steiner)	4 mm	5.1 mm	6.8 mm	5.6 mm	+1.7 mm
	1.NB (degrees)	(Steiner)	25^o^	20.2^o^	17.4^o^	21.2^o^	-2.8^o^
	1-NB (mm)	(Steiner)	4 mm	3.7 mm	4.3 mm	5 mm	+0.6 mm
	- Interincisal angle	(Downs)	130^o^	135.4^o^	140.7^o^	140^o^	+5.3^o^
	1-APo	(Ricketts)	1 mm	3.9 mm	2.2 mm	2.9 mm	-1.7 mm
Profile	Upper lip - S-line	(Steiner)	0 mm	-4 mm	-3.2 mm	-2.8 mm	-0.8 mm
	Lower lip - S-line	(Steiner)	0 mm	1.6 mm	-0.4 mm	-0.4 mm	-2.0 mm

## TREATMENT PLAN

The treatment plan consisted in performing rapid maxillary expansion followed by maxillary protraction with a facemask.^12^ Extraction of the mandibular canines would be performed to correct the negative overjet. Thereafter, Roth preadjusted appliances would be used, associated with Class III and anterior vertical elastics to complete correction of the anteroposterior and vertical discrepancies, respectively.

## TREATMENT PROGRESS

**Figure 5:**
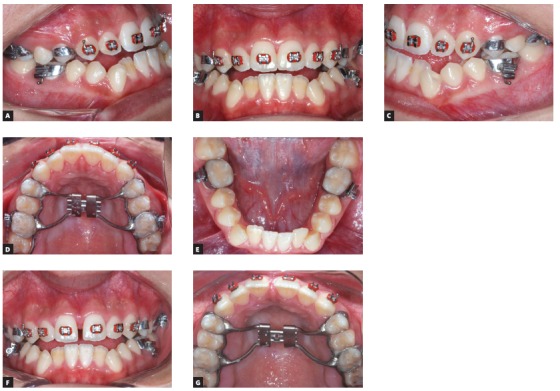
Installation of Hyrax and fixed orthodontic appliances (A-E) and expander activation based on Liou’s protocol (F-G).^13^

**Figure 6:**
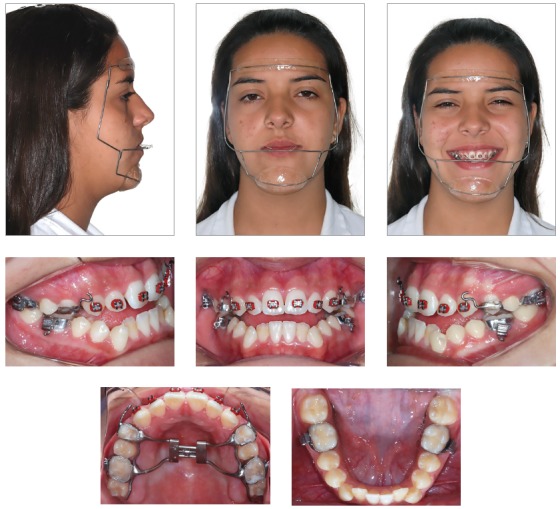
Facemask installation.

Treatment was initiated with rapid maxillary expansion, according to Liou’s protocol,^13^ which consists in activating the expander 1 mm per day, during 5 days, followed by closing the expander 1 mm per day, during 5 additional days. This procedure was repeated for three times (Fig 5). After the expansion, a facemask was installed to protract the maxilla, recommended to be used for 12 hours a day, with a force of 400g (Fig 6).

**Figure 7:**
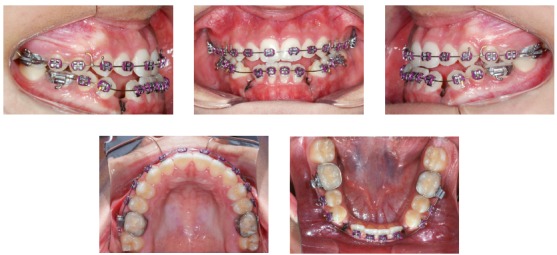
Intraoral photos of mandibular canine extractions.

**Figure 8:**
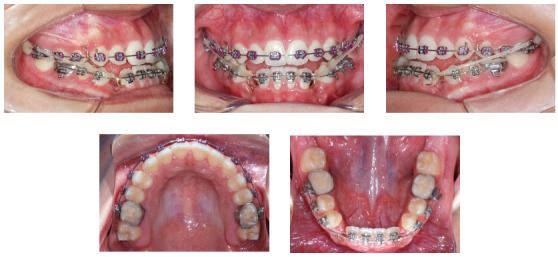
Mandibular anterior retraction and Class III elastics.

**Figure 9:**
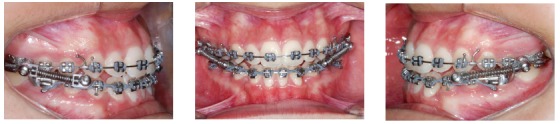
Mandibular anterior retraction and Class III mechanics with CS 2000 spring (DynaFlex, MO, USA).

Despite the efforts, there was only small maxillary protraction, positioning the incisors in an edge to edge relationship (Fig 7). Therefore, because of the persisting Class III anteroposterior relationship and the mandibular anterior crowding, the mandibular canines were extracted. At this time, Roth preadjusted appliances installation was completed. Leveling and alignment proceeded with 0.014 and 0.016-in NiTi archwires, followed by 0.016, 0.018 and 0.020-inch stainless steel archwires, with a hook on the distal of the mandibular lateral incisors, to engage Class III elastics, used for 18 hours a day, with 200g of force (Figs 7 and 8). Subsequently, rectangular 0.018 x 0.025-in archwires were installed to retract the mandibular incisors and to control torque during the use of Class III CS2000 springs (DynaFlex, MO, USA) or elastics (Fig 9). A chin-cup was used during sleeping hours to redirect mandibular growth, during treatment. After retraction of the mandibular incisors, vertical elastics were used to improve interdigitation. The total treatment time was of 3 years and 3 months.

After fixed appliances removal, a Hawley plate was installed in the maxillary arch, and recommended to be used 20 hours a day during 6 months; and night time use only, during the following 6 months. In the mandibular arch, a fixed first premolar-to-first premolar retainer was bonded on each tooth and recommended to be used for 3 years. The chin-cup was recommended to be used at night, as active retention, until the end of growth, which is approximately at age 20.^14^ ^,^ ^15^

## TREATMENT RESULTS

**Figure 10:**
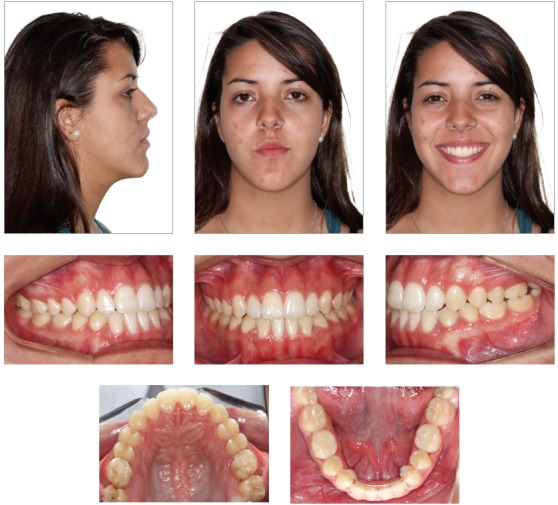
Final extra- and intraoral photographs.

**Figure 11:**
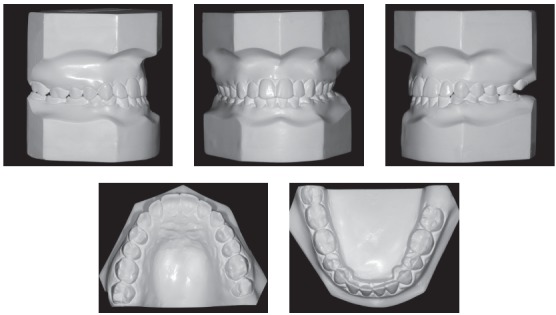
Final dental models.

The facial profile improved, showing passive lip seal and improvement of the zygomatic prominence (Fig 10). Consequent to crossbite and anterior open bite corrections, there was significant improvement of the smile esthetics (Figs 10 and 11).

**Figure 12:**
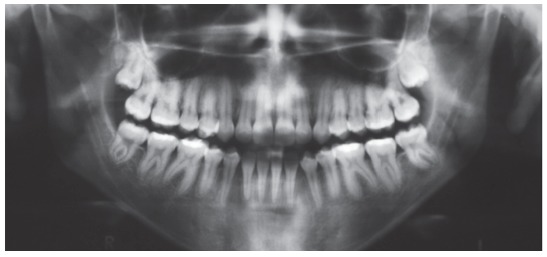
Final panoramic radiograph.

Due to slight maxillary protrusion and slight relative mandibular retrusion, there was improvement of the basal anteroposterior relationship, with reduction of the convexity angle (Fig 12 and Table 1). The maxilla had small anterior displacement provided by the facemask, Class III CS2000 springs and elastics, which were the factors that greatly contributed to correct the problem. The mandible experienced relative retrusion. 

**Figure 13:**
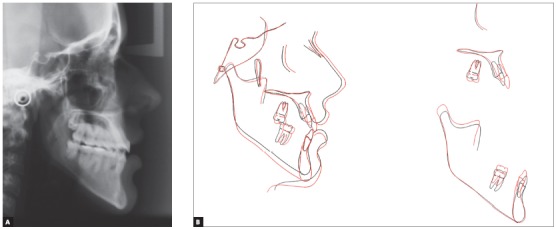
Final cephalometric radiograph and superimposition of initial (black) and final (red) tracing.

There was correction of the open and anterior cross-bites, the maxillary incisors were palatally tipped and protruded and the mandibular incisors were lingually tipped and slightly protruded (Figs 10 to 13, and Table 1). The mandibular first premolars replaced the canines and were positioned in Class I relationship with the maxillary canines, and the molars presented Class III relationship due to extractions of the mandibular canines (Fig 12).

**Figure 14:**
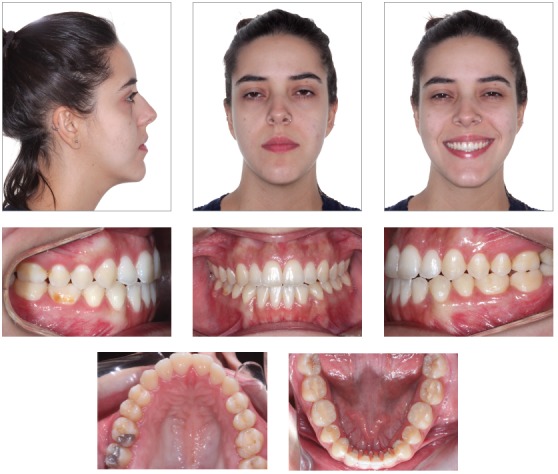
Seven-years posttreatment extra- and intraoral photographs.

**Figure 15:**
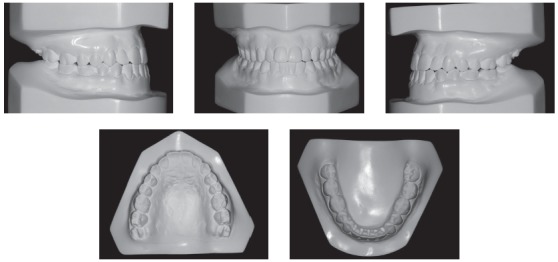
Seven-years posttreatment dental models.

**Figure 16:**
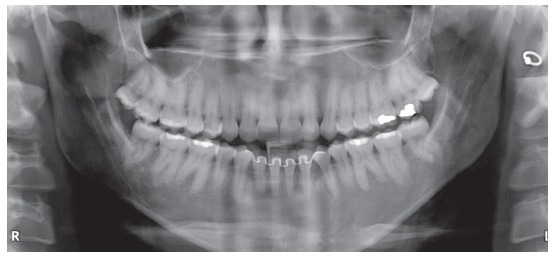
Seven-years posttreatment panoramic radiograph.

**Figure 17:**
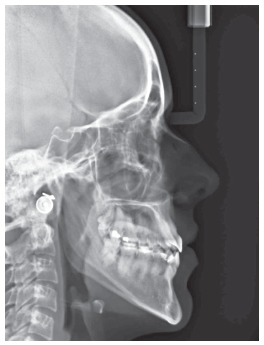
Seven-years posttreatment cephalogram.

Treatment remained fairly stable 7 years posttreatment, with the patient presenting good facial esthetics and occlusal relationships (Figs 14-17 and Table 1). The overjet and overbite are still positive and the transverse relationship is very satisfactory. All teeth are in contact and the third molars are present (Fig 16).

**Figure 18:**
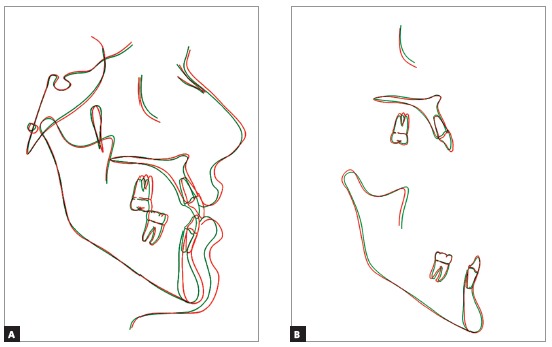
Superimposition of final (red) and long-term posttreatment (green) cephalometric tracings.

The tracings superimposition show that the anteroposterior basal discrepancy continued to improve (Fig 18 and Table 1). The maxillary incisors had slight palatal tipping and the mandibular incisors had slight labial tipping and protrusion. Molar relationship remained quite stable.

## FINAL CONSIDERATIONS

The initial maxillary expansion was able to improve the transverse deficiency of the maxilla. However, maxillary protraction, following Liou’s expansion protocol produced only slight improvement in the anteroposterior position of the maxilla (Table 1 and Fig 10). Perhaps patient’s age was a bit advanced.^16^ ^,^ ^17^ Besides, not every patient responds very favorably to maxillary protraction.^17^

There was relative retrusion of the mandible, which was probably due to retraction of the mandibular incisors and also to the effects of the CS2000 spring and Class III elastics^2^ ^,^ ^18^ ^-^ ^20^ (Figs 8, 9 and Table 1). The association of a slight maxillary protraction and mandibular retrusion produced improvement of the Class III anteroposterior relationship and decreased profile concavity.^19^ Despite the accentuated vertical growth pattern, the orthodontic mechanics did not produce a clockwise mandibular rotation. Probably the extraction mechanics helped in the vertical control.^21^

The negative overjet improved due to maxillary incisor protrusion and mandibular incisor lingual tipping during retraction (Table 1). Although the maxillary incisors were protruded, they also experienced palatal tipping. This demonstrates that there was excessive palatal resistant torque during Class III elastics/spring mechanics.^2^ ^,^ ^22^ A positive overbite was obtained consequent to extrusion of the mandibular incisors with the use of Class III elastics and vertical anterior elastics in the finishing procedures.^2^ ^,^ ^18^ ^,^ ^22^ ^,^ ^23^

The dentoskeletal changes provided improvement of the soft tissue profile, causing slight protrusion of the upper lip and retrusion of the lower lip, which contributed to establish a passive lip seal (Table 1 and Fig 10).

It was felt that a compensatory orthodontic treatment could provide satisfactory results in this patient because her facial esthetics was not significantly compromised and more importantly, because the patient and her parents did not want to undergo surgery. Perhaps an orthodontic-surgical approach would provide a better result. However, the patient and her parents were very satisfied with the obtained results.

The option of extracting the mandibular canines was taken because it would require less anchorage reinforcement to retract the anterior teeth. One can visualize that the first mandibular premolars were almost in a Class I relationship with the maxillary canines (Figs 1 and 7). Therefore, extracting the mandibular canines would only require incisor retraction and slight improvement of the anteroposterior discrepancy to obtain good anterior relationship. The first mandibular premolars would then replace the mandibular canines. There are no static or functional implications with this procedure.^5^

Evidently this treatment option also required great patient compliance in using the facemask and Class III elastics. The patient was not an excellent complier, but was satisfactory. This is the reason for the CS2000 spring have been used, especially in a time when the patient was already tired of using the Class III elastics. However, considering the obtained results, she performed well. After fixed appliances removal, she was instructed to use a chin-cup during the sleeping hours until the end of growth.^24^ However, she did not use it for a long time.

Despite her little compliance with posttreatment active retention, treatment has demonstrated to be very stable after 7 years (Figs 14 to 18). Her maxillary third molars erupted, but without antagonists. If they were overerupted in the next follow-ups, they would need to be extracted.

A detailed diagnosis has to be performed to provide good treatment results that also satisfies the patient and parents’ needs. A thorough analysis of the occlusal, skeletal and soft tissue components has to be performed to provide the adequate treatment for each individual situation.
